# The predictive value of the renal resistive index for contrast-induced nephropathy in patients with acute coronary syndrome

**DOI:** 10.1186/s12872-019-1017-3

**Published:** 2019-02-11

**Authors:** Zheng-rong Xu, Jun Chen, Yuan-hui Liu, Yong Liu, Ning Tan

**Affiliations:** 1 Southern Medical University , Guangzhou, 510515 China; 2 Department of Cardiology, People’s Hospital of Baoan Shenzhen, Shenzhen, 518100 China; 3 Department of Cardiology, Guangdong Cardiovascular Institute, Guangdong Provincial Key Laboratory of Coronary Heart Disease Prevention, Guangdong Provincial People’s Hospital, Guangdong Academy of Medical Sciences, Guangzhou, 510080 China

**Keywords:** Contrast-induced nephropathy, CIN, Renal resistive index, RRI

## Abstract

**Background:**

Percutaneous coronary intervention (PCI) has been associated with contrast-induced nephropathy (CIN) at a rate that varies depending on the patient’s risk factors. This study was conducted to evaluate the predictive value of the renal resistive index (RRI) for CIN in patients with acute coronary syndrome (ACS) undergoing PCI.

**Methods:**

This prospective study enrolled 146 consecutive patients with ACS. Renal Doppler ultrasound examinations to measure RRI were performed pre-PCI and at 1 h and 24 h after PCI. The primary endpoint was CIN, defined as a relative (≥25%) or absolute (≥0.5 mg/dL; 44 μmol/L) increase in serum creatinine from baseline within 48 h after contrast exposure.

**Results:**

CIN was identified in 31 patients (21.2%); however, none of the patients required haemodialysis. Compared to patients without CIN, higher RRIs were observed at 1 h (0.71 ± 0.05 vs. 0.65 ± 0.06, *p* < 0.05) and 24 h (0.70 ± 0.05 vs. 0.66 ± 0.06, *p* < 0.05) post-procedure in patients with CIN. The RRI rose transiently from baseline (0.68 ± 0.05) to 1 h (0.71 ± 0.05) and then tended to decline at 24 h (0.70 ± 0.05). A receiver operating characteristic curve analysis showed that the pre-procedure RRI was a powerful predictive indicator of CIN (area under the curve = 0.661, *p* = 0.006). The best cutoff value was 0.69 with 67.7% sensitivity and 67% specificity. Besides hyperuricemia and chronic kidney disease, the multivariate logistic regression analysis revealed that a high baseline RRI (≥0.69) was a significant predictor of CIN (odds ratio = 4.445; 95% confidence interval: 1.806–10.937; *p* = 0.001).

**Conclusions:**

A high pre-procedural RRI appears to be independently predictive of CIN in patients with ACS undergoing PCI.

## Background

Percutaneous coronary intervention (PCI) is associated with contrast-induced nephropathy (CIN) rates that vary between 0 and 24% depending on a patient’s risk factors [1]. Despite the fact that most cases are transient and reversible, CIN is associated with significant morbidity and mortality. Therefore, prevention and early diagnosis of CIN are extremely important. Currently, the diagnosis of CIN still depends on serum creatinine monitoring, which is a late marker of acute kidney injury (AKI). Several new potential serum and urinary biomarkers of AKI have been reported. Nevertheless, as solely fascinating findings at the basic science level, they remain far from clinical utility [2].

The renal resistive index (RRI) is one of the most sensitive parameters in renal pathology and reflects alterations in renal plasma flow. The RRI has been proven clinically useful for assessing renovascular hypertension, determining the prognosis for renal allografts and calculating AKI risk in the intensive care unit [3, 4]. Contrast medium (CM)-induced alterations in renal microvascular haemodynamics are considered to be one of the mechanisms in the development of CIN [1]. However, few studies have focused on the relationship between RRI and CIN. Hetzel et al. revealed that RRIs rose transiently after the administration of CM and then decreased progressively to baseline values [5]. Thus, we hypothesized that RRI is a good marker for determining the risk of developing CIN. Additionally, we considered that persistently elevated RRIs after CM exposure could indicate the possibility of contrast-induced AKI (CI-AKI).

## Methods

### Population selection

This prospective study on the predictive value of RRI for CIN (The Science and Technology Program of Guangzhou, project NO.201510010190) was conducted at the Affiliated Baoan Hospital of Shenzhen, Southern Medical University (People’s Hospital of Baoan District) between October 2015 and January 2017. We enrolled all consecutive patients with ST-segment elevation myocardial infarction (STEMI) or non-ST-elevation acute coronary syndrome (NSTE-ACS) undergoing PCI at our centre. The exclusion criteria were as follows: (a) cardiogenic shock prior to admission; (b) insertion of an intra-aortic balloon pump (IABP); (c) chronic peritoneal or haemodialysis treatments; (d) exposure to CM within the previous 7 days; (e) severe obesity (body mass index> 40 kg/m^2^) and (f) stenosis in either renal artery. The study protocol was approved by the ethics committee of our hospital, and all enrolled patients provided written informed consent to participate in the study.

### Study protocol and risk calculation

Blood samples were obtained from all patients at the time of hospital admission. The tests comprised baseline standard laboratory panels including serum creatinine (SCr) levels. SCr was also measured daily for 2 days after CM exposure. The estimated glomerular filtration rate (eGFR) was calculated using the four-variable Modification of Diet in Renal Disease equation for Chinese patients [6]. Other clinical parameters, such as troponin I, creatine kinase MB and N-terminal pro-brain natriuretic peptide (NT-proBNP) were evaluated as part of our standard clinical care.

### PCI procedures and medications

According to guidelines, patients with STEMI were treated with urgent mechanical reperfusion (primary PCI). Variable revascularization strategies were used according to a risk stratification model that was instituted for patients with NSTE-ACS. These strategies included immediate invasive treatment for patients considered to be extremely high risk, early invasive treatment for those considered to be high risk and a routine invasive approach (< 72 h) for those considered to be low and moderate risk. The invasive procedures were performed by an experienced interventional cardiologist. Non-ionic, low-osmolality CM (Iopamiron, 370 mg I/mL) was used in all patients. The CM volume was determined by the interventional cardiologist according to the patient’s needs. Hydration with intravenous 0.9% saline was started at a rate of 1 mL/kg/h during the procedure and continued until 6–12 h afterward. All patients received a loading dose of dual anti-platelet therapy (aspirin 300 mg plus clopidogrel 600 mg for the immediate invasive strategy or 300 mg aspirin plus 300 mg clopidogrel if the invasive intervention was delayed). This was followed by aspirin 100 mg plus clopidogrel 75 mg daily. Patients were treated with angiotensin-converting enzyme inhibitors, beta-blockers and statins according to standard guidelines. The Glycoprotein IIb/IIIa inhibitors were administered at the cardiologist’s discretion.

### Renal ultrasound Doppler examination

All patients underwent ultrasound examinations with RRI determinations before and at 1 and 24 h after the procedure. According to the recommendations of Granata et al. [3], colour Doppler evaluations were performed in three different areas of each kidney (upper, mid and lower poles) and were focused on the arcuate or interlobular arteries. The gains were set to the highest possible, with the lowest filters and low pulse repetition frequencies [3]. The patients were placed in the supine position and rested for 10 min before the examination. Throughout the assessment, we obtained RRI directly. Echocardiography was subsequently performed to determine the left ventricular ejection fraction (LVEF). The ultrasound examinations were performed by one experienced physician using a Philips CX 50 (Philips, Amsterdam, Netherlands) with a probe (2–5 MHz). In general, the examinations were performed during the primary PCI preparation period, and the whole examination took approximately 5 min.

### Primary endpoint

The primary endpoint was the onset of CIN, classically defined by a relative (≥25%) or absolute (≥0.5 mg/dL; 44 μmol/L) increase in serum creatinine from baseline within 48 h after CM exposure, after ruling out other factors that could cause nephropathy [7].

### Statistical analysis

Continuous variables are expressed as means ± standard deviations and compared using the *t* test or Wilcoxon rank-sum test. Categorical variables are presented as frequencies or percentages and were compared using the Pearson Chi-Square test. The optimum cutoff point for RRI to predict CIN was established according to the receiver operating characteristic (ROC) curve analysis. Multivariate logistic regression was performed using a forward stepwise selection process to evaluate the independent values of RRI as categorical variables (based on the cutoff values) for CIN. Variables for inclusion were carefully chosen, including those that were considered clinically relevant or those that showed a significant outcome (*p* < 0.1) in the univariate model. SPSS software (version 19.0; SPSS Inc., Chicago, IL, USA) was used for all the analyses. All statistical tests were two-tailed and statistical significance was accepted at *p* < 0.05.

## Results

**Fig. 1:**
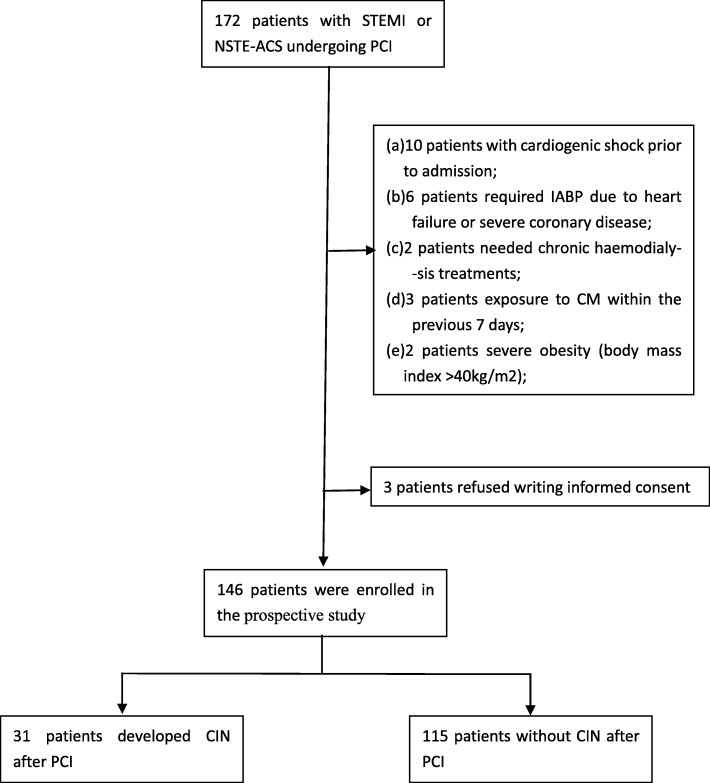
Flow chart showing the study population selection process. A total of 172 patients with ACS underwent PCI between October 2015 and January 2017; 23 patients were excluded based on the following criteria: **a** 10 patients with cardiogenic shock prior to admission; **b** 6 patients required IABP due to heart failure or severe coronary disease; **c** 2 patients required chronic haemodialysis; **d** 3 patients were exposed to CM within the previous 7 days; **e** 2 patients with severe obesity (body mass index> 40 kg/m^2^. Besides, 3 patients declined to sign the written informed consent. Finally, 146 patients were enrolled and analysed in this prospective study. ACS: acute coronary syndrome; PCI: percutaneous coronary intervention; IABP: intra-aortic balloon pump; CM: contrast medium

A total of 172 patients with ACS underwent PCI at our centre between October 2015 and January 2017; of these, 23 patients were excluded according to the exclusion criteria and 3 patients declined to sign the written informed consent (Fig. 1). Finally, 146 patients were enrolled and analysed in this prospective study, including 65 patients with STEMI (44.5%) and 81 with NSTE-ACS (55.5%). There were 31 women and 115 men with a mean age of 56.6 ± 12.4 years. The mean eGFR was 92.4 ± 28.0 mL/min/1.73 m^2^, which included 15 patients with moderate and 4 with severe renal impairments. The percentage of patients with complicating conditions including hypertension, diabetes, hyperlipidaemia and hyperuricemia were 30.8, 47.9, 50.7 and 22.6% respectively. Fifty-six patients (38.4%) had multi-vessel disease (more than triple-vessel) and the left anterior descending branch (LAD) was the target vessel in 63 (43.2%). Transient peri-procedural hypotension occurred in 6 patients (4.1%) and none required IABP placement.

The mean pre-procedure SCr value was 84.5 ± 37.3 μmol/L, while at 48 h post-procedure, the mean SCr value rose to 90.4 ± 38.5 μmol/L. CIN developed in 31 patients (21.2%), but none required haemodialysis. There were no significant differences in the rates of diabetes, hypertension and hyperlipidaemia or differences in other pre-procedure parameters including age, body mass index, CM and SCr between the CIN and non-CIN groups. Nevertheless, patients with CIN were characterized by higher hydration volume, higher incidence of hyperuricemia, and lower eGFRs. Moreover, a trend toward lower LVEFs and the LAD being the more likely target vessel was observed in patients with CIN (Table 1).

**Table 1:** Baseline clinical characteristic of patients with and without CIN

Variable	CIN (+) *n* = 31	CIN (−) *n* = 115	*P* value
Women	9 (29)	22 (19.1)	0.232*
Age (years)	57.1 ± 14.2	56.4 ± 12.0	0.770
BMI (kg/m^2^)	24.5 ± 4.6	24.5 ± 3.7	0.975
Smoking, n (%)	12 (38.7)	34 (29.6)	0.331
Hypertension, n (%)	12 (38.7)	58 (50.4)	0.246*
Diabetes mellitus, n (%)	7 (22.6)	38 (33.0)	0.263*
Hyperlipidaemia, n (%)	19 (61.3)	55 (47.8)	0.183*
Hyperuricemia, n (%)	12 (38.7)	21 (18.3)	0.016*
CKD, n (%)	8 (25.8)	10 (8.7)	0.010*
Pre-procedure SCr (μmol/L)	98.7 ± 39.8	86.0 ± 37.7	0.101
Pre-procedure eGFR (ml/min/1.73m^2^)	81.3 ± 32.6	93.1 ± 27.5	0.045
< 30 n (%)	2 (6.5)	2 (1.7)	
≥30, < 60 n (%)	6 (19.4)	9 (7.8)	
≥60 n (%)	23 (74.2)	104 (90.4)	
CHF n (%)	5 (16.1)	24 (20.9)	0.557*
Hypotension, n (%)	0 (0)	6 (5.2)	0.194*
LVEF(%)	50.0 ± 9.0	53.9 ± 10.0	0.053
Diagnosis, n (%)			0.615*
UA	5 (16.1)	26 (22.6)	
NSTEMI	10 (32.3)	40 (34.8)	
STEMI	16 (51.6)	49 (42.6)	
Diseased vessels	2.0 ± 1.0	2.1 ± 0.8	0.492
MVD, n (%)	12 (38.7)	44 (38.3)	0.964*
LAD target vessel, n (%)	18 (58.1)	45 (39.1)	0.059*
Contrast volume (mL)	124 ± 43	121 ± 32	0.686
Contrast volume/kg (mL/kg)	1.9 ± 0.8	1.8 ± 0.6	0.650
24 h hydration volume (mL)	2180 ± 681	1868 ± 701	0.029
Haemoglobin (g/L)	135.1 ± 20.7	132.7 ± 19.4	0.548
Homocysteinemia (μmol/L)	17.3 ± 4.8	13.3 ± 3.7	0.000
SBP pre-procedure (mmHg)	133 ± 16.5	126 ± 21.6	0.116
DBP pre-procedure (mmHg)	81.3 ± 11.7	80.0 ± 15.4	0.671

*using the *Pearson Chi-Square* test

*BMI* Body mass index, *CKD* Chronic kidney diseases, *UA* Unstable angina, *NSTEMI* Non-ST segment elevated myocardial infarction, *STEMI* ST segment elevated myocardial infarction, *CHF* Congestive heart failure, *LAD* Left anterior descending artery, *LVEF* Left ventricular ejection fraction, *MVD* Multi-vessel disease, *SBP* Systolic blood pressure, *DBP* Diastolic blood pressure, *SCr* Serum creatinine, *eGFR* Estimated glomerular filtration rat

RRI showed no significant differences before and after CM exposure in all of the patients, and also in the non-CIN group. Nevertheless, a much higher RRI value was observed at 1 h and 24 h after CM exposure in the patients with CIN (Table 2). An analysis of the CIN group showed that, compared to baseline, the RRI rose significantly at 1 h (0.71 ± 0.05 vs 0.68 ± 0.05, respectively; *p* = 0.030), but there was no significant elevation at 24 h (0.70 ± 0.05 vs 0.68 ± 0.05, respectively; *p* = 0.071) (Table 2).

**Table 2:** Comparisons between and within groups in terms of RRI at different times

Variable	RRI		
	Total	CIN (+)	CIN (−)
Pre-procedure	0.66 ± 0.05	0.68 ± 0.05^a^	0.66 ± 0.05
1 h after procedure	0.66 ± 0.07	0.71 ± 0.05^ab^	0.65 ± 0.06^b^
24 h after procedure	0.67 ± 0.06	0.70 ± 0.05^b^	0.66 ± 0.06^b^

^a^*P* < 0.05 within groups; ^b^*P* < 0.05 between groups

*RRI* Renal resistive index, *CIN* Contrast-induced nephropathy

The ROC curve analysis indicated that the pre-procedure RRI was a powerful predictor of CIN (area under the curve = 0.661, 95% confidence interval (CI) 0.549–0.772; *p* = 0.006). Next, we determined that the best cutoff value based on the Youden index was 0.69, with 67.7% sensitivity and 67% specificity (Fig. 1).

The selected variables and results of the multivariate logistic regression analysis are presented in Table 3. The multivariate logistic regression analysis revealed that a pre-procedure RRI ≥0.69 was positively associated with CIN (odds ratio (OR) = 4.445; 95% CI 1.806–10.937; *p* = 0.001). Other significant factors included hyperuricemia (OR 2.607; 95% CI 1.007–6.749; *p* = 0.048) and chronic kidney disease (CKD; OR 3.163; 95% CI 0.999–10.013; *p* = 0.050) (Table 3).

**Table 3:** Univariate and multivariate analyses of risk factors for CIN

Variable	OR	95%CI	p
Univariate analysis			
LAD target vessel	2.154	0.962–4.821	0.062
LVEF	0.962	0.924–1.001	0.056
CKD	3.652	1.299–10.265	0.014
Hyperuricemia	2.827	1.192–6.706	0.018
24 h hydration volume (mL)	1.001	1.000–1.001	0.032
eGFR	0.986	0.972–1.000	0.048
RRI pre-procedure ≥0.69	4.093	1.727–9.698	0.001
Contrast volume	1.003	0.992–1.014	0.629
Contrast volume/kg	1.180	0.642–2.168	0.593
Multivariate analysis			
RRI pre-procedure ≥0.69	4.445	1.806–10.937	0.001
Hyperuricemia	2.607	1.007–6.749	0.048
CKD	3.163	0.999–10.013	0.050

*LAD* Left anterior descending artery, *CKD* Chronic kidney diseases, *LVEF* Left ventricular ejection fraction, *eGFR* Estimated glomerular filtration rate, *RRI* Renal resistive index

## Discussion

The present study was designed to identify the risk factors for CI-AKI relative to the RRI, which was a novel undertaking. Our results indicate that the renal vascular bed pathology contributes to an increase in the incidence of CI-AKI in patients with ACS who are referred for PCI.

RRI is calculated as (Vsyst-Vdiast)/Vsyst [8] and reflects the resistance in the renal vascular beds. RRI is affected by renal interstitial disease or vascular compliance as well as extra-renal factors such as blood pressure, heart rate and rhythm, age and significant aortic valve stenosis [9]. To date, it is one of the most sensitive parameters for kidney disease and can provide useful information about the pathophysiology of renal diseases in both native and transplanted kidneys [3]. However, there has been little focus on the relationship between RRI and CI-AKI. Wybraniec et al. firstly revealed that a high pre-procedure RRI value may be a useful and novel risk factor for CI-AKI [10]. However, there were some limitations in that study, such as the absence of patients with pre-existing renal function impairment, and only 9 patients with evidence of CI-AKI onset. In our study, we maintained the same viewpoint as Wybraniec and found that the RRI after contrast administration was higher in patients with CI-AKI and was likely to be higher at baseline. Normal RRI values in adults range from 0.47 to 0.70. The patients enrolled in our study could be considered to have ‘high-normal’ RRI values, which may mainly affectedly by age and vascular bed atherosclerotic, appropriately depending on these high rates of concomitant diseases entity (e.g., diabetes, CKD) [11]. Nevertheless, no significant differences were observed between the two groups with respect to extra-renal factors, and peri-procedural hypotension occurred in only a small number of patients. Therefore, a higher prone baseline RRI value in the CIN group may mainly indicate that the renal vascular resistance tended to be higher, either for renal interstitial disease or vascular compliance. Thus, patients with CIN had worse baseline renal function or more severe vascular bed atherosclerosis, which could explain why the RRIs after CM administration rose significantly in the CIN group and the patients with a relatively high baseline RRI were more prone to developing CI-AKI. Our study showed that patients with a high RRI (≥0.69) were more likely to develop CI-AKI.

In clinical practice, most cases of CI-AKI are reversible and seldom are these patients referred for haemodialysis. There is a similar alteration in RRI values following CM exposure. Hetzel et al. showed that RRI increased significantly within minutes after the administration of intravenous CM and then decreased progressively to baseline [5]. This could explain why RRI, after CM exposure in the non-CIN group, was not significantly different in comparison to the baseline standard value in our study. RRI rose transiently after CM exposure in general; nevertheless, this RRI alteration seemed to be prolonged and even lasted more than several hours in the CIN group. We think that the prolonged alteration might be relevant in patients with CI-AKI that have a delayed vascular response to CM [12]. Although subsequent changes in RRI were not recorded, the SCr returned to baseline and the absence of oliguria or anuria suggested that these changes were functional and reversible. This provides important evidence for the hypothesis that CI-AKI is related to vasoconstriction in the renal vessel beds, especially after CM exposure, which causes renal medullary hypoxia.

The multivariate logistic regression analysis showed that a high baseline RRI (≥0.69), hyperuricemia and CKD accurately predicted CI-AKI. LAD as a target vessel, LVEF, eGFR and 24-h hydration volumes were thought to be risk factors based solely on the univariate analysis, but these factors were excluded after the multivariate analysis. It is no doubt that patients with baseline CKD are high-risk for CI-AKI. Hyperuricemia has been demonstrated to be independently associated with an increased risk of CKD in cross-sectional studies [13]. In contrast to previous studies, our results excluded risk factors such as age, contrast volume, diabetes mellitus and a history of congestive heart failure (CHF) [14]. There might be several reasons why these differences were related to our unique study design. First, the mean age of the patients was 56.6 ± 12.4 years; therefore, they might have had diabetes for only a short time and atherosclerosis may not yet have developed. On the other hand, RRI was also thought to be closely related to atherosclerosis which may cause a confusing effect. [11]. Second, we enrolled a large number of patients with STEMI, most of whom required primary PCI and were administered relatively small volumes of CM (≤100 mL, in general). Finally, we did not discriminate between pre-existing CHF and new-onset CHF caused by acute myocardial infarction, which may have also been a confounder.

The present study had several limitations. First, it was a single-centre study, and we enrolled a relatively small number of patients with ACS. Second, we may have overestimated the true incidence of CI-AKI. Some patients may have easily met the diagnostic criteria for CIN (a relative rise of SCr ≥25%) after CM exposure, especially when the baseline SCr was relatively low. Moreover, SCr values could have been affected by haemodynamic changes in the setting of acute myocardial infarction. Third, although we were committed to minimizing errors (colour Doppler was performed in three different areas of the kidney and was focused on the arcuate/interlobular arteries), the RRI values may have been affected by the view angle and patient positioning. Thus, employing an invasive Doppler flow wire might be an ideal alternative method of measuring RRI [12].

## Conclusions

The current study showed that a relatively high pre-procedural RRI appears to be an independent predictor of CIN in patients undergoing PCI and that preoperative RRI assessment facilitates stratification of risk for CI-AKI among patients referred for coronary angiography. A significantly increased RRI after CM administration is suggestive of developing CI-AKI; however, the absolute value of the increase is still unknown and may be our focus of study in the future. Moreover, an analysis of intrarenal arterial Doppler flow profiles constitutes a favourable means of investigating the pathophysiologic mechanisms of CI-AKI.

## Abbreviations

ACS: Acute coronary syndrome
AKI: Acute kidney injury
CHF: Congestive heart failure
CIN: Contrast-induced nephropathy
CKD: Chronic kidney disease
CM: Contrast medium
eGFR: Estimated glomerular filtration rate
IABP: Intra-aortic balloon pump
LAD: Left anterior descending artery
LVEF: Left ventricular ejection fraction
PCI: Percutaneous coronary intervention
ROC: Receiver operating characteristic
RRI: Renal resistive index
SCr: Serum creatinine
